# Associations between Abdominal Trunk Muscle Weakness and Future Osteoporotic Vertebral Fracture in Middle-Aged and Older Adult Women: A Three-Year Prospective Longitudinal Cohort Study

**DOI:** 10.3390/jcm11164868

**Published:** 2022-08-19

**Authors:** Satoshi Kato, Satoru Demura, Kazuya Shinmura, Noriaki Yokogawa, Yuki Kurokawa, Ryohei Annen, Motoya Kobayashi, Yohei Yamada, Satoshi Nagatani, Hidenori Matsubara, Tamon Kabata, Hiroyuki Tsuchiya

**Affiliations:** Department of Orthopaedic Surgery, Graduate School of Medical Sciences, Kanazawa University, 13-1 Takara-machi, Kanazawa 920-8641, Japan

**Keywords:** abdominal trunk muscle strength, older adult women, prospective study, osteoporotic vertebral fracture, risk factor

## Abstract

Potential risk factors associated with future osteoporotic vertebral fracture (OVF) were prospectively investigated in middle-aged and older adult women. We enrolled 197 female patients aged ≥50 years who were scheduled to undergo surgery for lower-extremity degenerative diseases. Patient anthropometric and muscle strength measurements, a bone mineral density measurement of the lumbar spine (L-BMD), and full-spine standing radiographs to examine the presence of old OVFs and spinopelvic sagittal parameters were obtained preoperatively. We evaluated 141 patients who underwent full-spine standing radiographs three years postoperatively to identify new OVFs. We excluded 54 patients who did not undergo a second radiographic examination and 2 with new traumatic OVFs. Univariate and multivariate analyses were performed to identify risk factors associated with new non-traumatic OVF occurrence. Ten (7.1%) patients developed new non-traumatic OVFs during the study period (fracture group). The fracture group had less abdominal trunk muscle strength, lower L-BMD, smaller sacral slopes, and larger pelvic tilt than the non-fracture group. The fracture group showed a higher prevalence of old OVFs preoperatively than the non-fracture group. Abdominal trunk muscle weakness, low L-BMD, and the presence of old OVFs were identified as significant risk factors for OVF occurrence. In middle-aged or older adult women, abdominal trunk muscle weakness, low L-BMD, and old OVFs were associated with future OVF.

## 1. Introduction

Osteoporotic vertebral fractures (OVFs), the most common type of fracture in older adults [1], have been described as the “hallmark of osteoporosis” [2]. A positive history of OVF strongly influences the likelihood of subsequent OVF and related mortality [3,4,5,6]. Fracture prevalence and incidence rates have been reported to increase with age; low bone mineral density (BMD) is a predictor of OVF [7]. However, the causes of OVF are often unclear. Non-traumatic vertebral fractures account for 83% of all vertebral fractures; these fractures are frequently asymptomatic and recurrent [8]. Fracture history has also been associated with an increased risk of subsequent fracture [9,10]. These circumstances suggest that there are important but poorly understood factors apart from BMD that increase the risk of developing OVF.

Neuromuscular aging-related changes, including the loss of lean muscle mass and a decline in muscle strength, have been well known [11]. The decline in muscle strength with aging is two to five times faster than that in muscle size in the lower extremities [12]. Two studies have reported that older adults have decreased lumbar extensor strength [13] and increased fatty infiltration of the trunk musculature [14]. Recent studies have investigated the relationship between spinal sagittal imbalance and the incidence of OVF [15,16,17]. Spinopelvic sagittal alignment plays an important role in the biomechanical adaptation of the pathological spine, particularly in the older adult population. A recent review of in vivo and computational modeling studies emphasized the importance of understanding spinal loading to better prevent and manage spinal disorders [18]. While the causes of OVFs are unclear, the neuromuscular function of the trunk muscles and spinopelvic alignment may play an important role in its pathogenesis.

In a previous study, we reported that abdominal trunk muscle weakness, older age, and low lumbar bone mineral density (L-BMD) were significant risk factors associated with the presence of OVFs in the lower thoracic and lumbar spine of middle-aged and older adult women [19]. However, a causal relationship could not be determined owing to the study’s cross-sectional design. Therefore, in this three-year prospective longitudinal cohort study, we aimed to identify the risk factors for future OVF occurrence among middle-aged and older adult women in relation to muscle strength and radiographic findings concerning spinopelvic alignment.

## 2. Materials and Methods

### 2.1. Ethics Statement

Our university hospital ethics committee approved this study (No. 2015-109). Written informed consent was obtained from each prospective participant before registration by the research physicians according to the Declaration of Helsinki.

### 2.2. Study Participants

**Figure 1:**
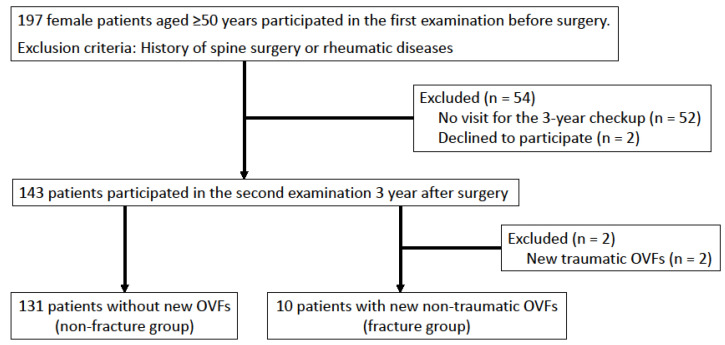
Study flowchart. The baseline study included 197 female patients aged ≥50 years who were scheduled to undergo surgery for lower-extremity degenerative diseases, of whom 54 were excluded from the study because they failed to attend the three-year postoperative checkup. Two patients were excluded because of traumatic OVF occurrence.

Between January 2016 and December 2018, the clinical data of 197 female patients aged ≥50 years who were scheduled to undergo surgery for degenerative diseases of the lower extremities at our hospital and agreed to participate in a preoperative examination were prospectively collected. Patients who had previously undergone spine surgery or had been diagnosed with rheumatic diseases were excluded from the study. Before surgery, the included study patients underwent physical measurements, full-spine standing radiographic examinations, and L-BMD measurements. Three years postoperatively, these patients underwent an additional full-spine standing radiographic examination. The presence of new OVFs was determined by comparing full-spine standing radiographs taken before surgery with those taken 3 years postoperatively. In this study, we defined traumatic OVF as a vertebral fracture due to an apparent trauma, such as a fall. Non-traumatic OVF was defined as a vertebral fracture that occurred without any particular injury mechanism. We categorized OVFs as traumatic or non-traumatic; patients with traumatic OVF were excluded from the analysis (Figure 1).

### 2.3. Evaluation

We obtained anthropometric measurements, including body height, weight, and mass index. Hand grip strength was measured using a dynamometer (TTM Dynamometer; Tsutsumi, Tokyo, Japan). Knee extensor muscle strength (KEMS) was measured using a hand-held dynamometer (μTas F-1; ANIMA Corp., Tokyo, Japan), and KEMS values were divided by body weight (N/kg). To measure KEMS, patients were seated on an elevated chair with their knees flexed at 90° and their feet off the floor. With the dynamometer placed on the anterior leg surface, 10 cm proximal to the malleoli, the patients were instructed to push against the dynamometer by straightening their knees [20]. Intra- and inter-rater reliabilities of the KEMS measurements using this method have been reported to be acceptable [20]. Right and left grip power and KEMS were measured once, and the higher strength value of each measurement was recorded. Abdominal trunk muscle strength (ATMS) was measured using an exercise device designed for abdominal trunk muscles (RECORE: Nippon Sigmax Co., Ltd., Tokyo, Japan). Muscle strength was measured twice, and the higher strength value was recorded. As previously described in detail [21], this device enables patients to perform strength measurement or strengthening exercises involving their abdominal trunk muscles while sitting without moving the trunk or load on the spine. A previous study reported that this device had excellent intra- and inter-rater reliabilities for measuring ATMS and that strengthening exercises using the device activate and increase diaphragmatic, abdominal, and pelvic floor muscle strength [22]. Locomotive syndrome, a condition of reduced mobility due to an impaired locomotive organ, was assessed using the 25-question Geriatric Locomotive Function Scale (GLFS-25) for each patient [23]. We also obtained each patient’s five-point numerical rating scale (NRS) score for back pain (from 0 = no pain to 4 = severe pain) from the results for the second question on the GLFS-25 [20]. L-BMD was measured with dual X-ray absorptiometry in posteroanterior projection (GE Lunar Prodigy, GE Healthcare, Madison, WI, USA) using standardized procedures and centralized quality control.

Based on full-spine standing radiographic findings, we determined the presence of OVF in the lower thoracic or lumbar spine and measured the sagittal spinal alignment. OVFs were defined as grades 1–3 fractures according to the Genant semiquantitative method, indicating at least a 20% loss in the height of the vertebral body [24]. Sagittal balance was assessed using the sagittal vertical axis [15,16,17]. Lumbar lordosis, pelvic incidence, sacral slope, and pelvic tilt were measured to determine the spinopelvic sagittal parameters [15,16,17]. Pelvic incidence minus lumbar lordosis was also calculated and evaluated as an important parameter for spinopelvic sagittal balance [25]. Spinopelvic parameter measurements using this method have been reported to be accurate and reliable [26].

According to the occurrence of non-traumatic OVFs in the three-year study period, the patients were divided into fracture and non-fracture groups. Clinical factors were compared between the two groups, including age, body mass index, hand grip strength, KEMS, ATMS, GLFS-25 score, NRS score for back pain, L-BMD, and radiographic findings of the presence of old OVFs and the spinopelvic sagittal alignment parameters, including the sagittal vertical axis, lumbar lordosis, pelvic incidence, sacral slope, pelvic tilt, and pelvic incidence minus lumbar lordosis.

### 2.4. Statistical Analysis

Continuous variables are expressed as means ± standard deviations, and ordinal variables are expressed as medians (interquartile ranges). A Shapiro–Wilk test was used to assess the normality of data distribution. Between-group differences in the continuous variables were examined using Student’s *t*-test for parametric data and a Mann–Whitney U test for nonparametric data. Categorical data are expressed as frequencies and percentages, and comparisons between groups were made using a chi-square test. To identify factors associated with the occurrence of new OVFs in the lower thoracic or lumbar spine over the three-year study period, a multiple logistic regression model was used to obtain adjusted odds ratios with 95% confidence intervals (CIs). Finally, a receiver operating characteristic (ROC) curve analysis was used to determine the optimal cutoff for the occurrence of OVF. SPSS version 19.0 for Windows (SPSS Inc., Chicago, IL, USA) software was used for all statistical analyses, with the level of statistical significance set at 0.05.

## 3. Results

In total, 143 study participants underwent a 3-year checkup and radiographic examination with full-spine standing radiography (follow-up rate, 72.6%; Table 1). The patients without a second examination, who were excluded from the study, were older than those with these examinations. However, there were no significant differences in BMI, L-BMD, or the presence of old OVFs. Two participants with new traumatic OVFs were excluded from the study. Finally, 141 female participants aged ≥ 50 years were included for evaluation (Figure 1).

A total of 17 (12.1%) study participants had old OVFs before surgery. Another 13 participants had a single old OVF (2, 1, 4, 3, 2, and 1 participants at T8, T11, T12, L1, L3, and L4, respectively), and four had multiple old OVFs. Additionally, 10 (7.1%) participants with a mean age of 67.5 years (range, 55–82) had developed new non-traumatic OVFs during the three-year study period; these patients were assigned to the fracture group. The remaining 131 participants without new OVFs were assigned into the non-fracture group. Seven of the ten participants in the fracture group had a single OVF (one, three, one, and two participants at T8, T11, T12, and L3, respectively), while the other three had multiple OVFs.

In the univariate analyses, ATMS and L-BMD values were significantly lower in the fracture group (Table 2). In addition, the fracture group had smaller sacral slope and larger pelvic tilt values than the non-fracture group. The prevalence of old OVFs in the fracture group was significantly higher than that in the non-fracture group (60% vs. 8.4%, respectively). In the multiple logistic regression analysis, weak AMTS, low L-BMD, and the presence of old OVFs were significant risk factors for the occurrence of new OVFs in the lower thoracic or lumbar spine (Table 3).

**Figure 2:**
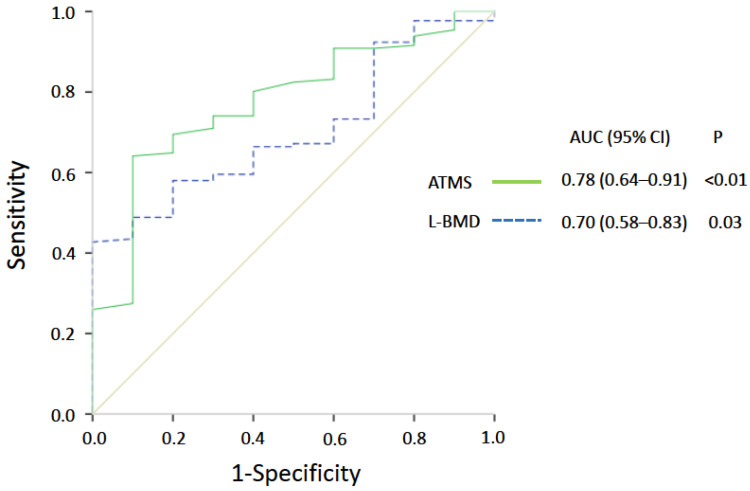
Receiver operating characteristic curves. The analysis revealed that the best cutoff points for ATMS and L-BMD were 4.0 kPa with an AUC of 0.78 and 1.11 g/cm^2^ with an AUC of 0.70, respectively. Abbreviations: AUC, area under the curve; ATMS, abdominal trunk muscle strength; CI, confidence interval; L-BMD, bone mineral density of the lumbar spine.

**Figure 3:**
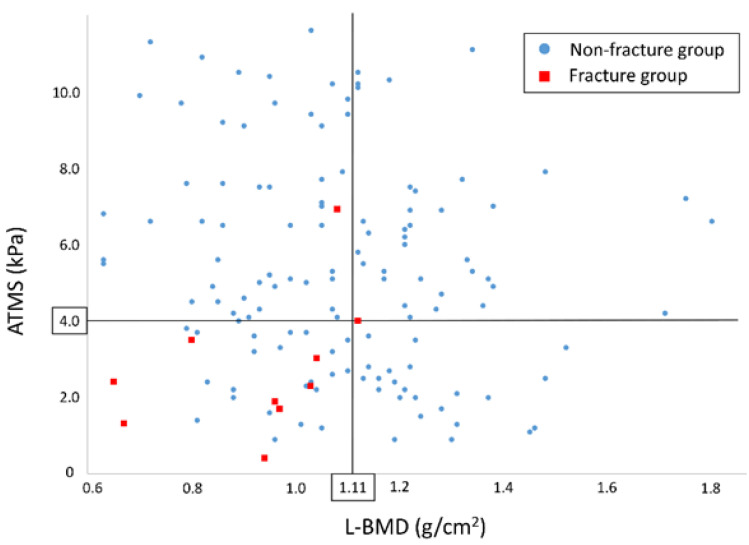
The distribution according to the patients’ AMTS and L-BMD at the first preoperative evaluation. The occurrence rate of new OVF was significantly higher in participants with AMTS values ≤ 4.0 kPa (16.1%, 9/56) than in those with AMTS values > 4.0 kPa (1.2%, 1/85, *p* = 0.001). It was also significantly higher in participants with L-BMD values ≤ 1.11 g/cm^2^ (11.0%, 9/82) than in those with L-BMD values > 1.11 g/cm^2^ (1.7%, 1/59, *p* = 0.031). Abbreviations: AMTS, abdominal trunk muscle strength; L-BMD, bone mineral density of the lumbar spine; OVF, osteoporotic vertebral fracture.

ROC analysis showed AMTS values ≤ 4.0 kPa (95% CI 0.643–0.909, *p* = 0.004, area under the curve 0.776; Figure 2) and L-BMD values ≤ 1.11 g/cm^2^ (95% CI 0.575–0.833, *p* = 0.032, area under the curve 0.704; Figure 2) best predicted the occurrence of OVF in the study cohort. Figure 3 shows the distribution of patients’ AMTS and L-BMD values at the first preoperative evaluation. The occurrence rate for new OVFs was significantly higher in patients with AMTS values ≤ 4.0 kPa (16.1%, 9/56) than in those with AMTS values > 4.0 kPa (1.2%, 1/85, *p* = 0.001). Similarly, the occurrence rate for new OVFs was significantly higher in participants with L-BMD values ≤ 1.11 g/cm^2^ (11.0%, 9/82) than in those with L-BMD values > 1.11 g/cm^2^ (1.7%, 1/59, *p* = 0.031).

## 4. Discussion

In this study, 10 (7.1%) patients developed new non-traumatic OVFs during the study period. These patients had lower ATMS and L-BMD, smaller sacral slope, larger pelvic tilt, and a higher prevalence of old OVFs at the initial examination preoperatively than the 131 patients who did not have new OVFs. The multivariate analysis showed that weak ATMS, low L-BMD, and the presence of old OVFs were risk factors for the occurrence of a new OVF. Low BMD and the presence of old OVFs are well-known predictors of OVF [3,4,7,15,27].

To the best of our knowledge, this is the first study to focus on the effect of ATMS on the future occurrence of OVF in middle-aged and older adult women and report muscle weakness as a novel risk factor for OVF occurrence. However, other muscle strength test results for grip power and KEMS and the potential for back pain to affect trunk muscle strength did not differ between the two groups.

A previous study reported that the device used in this study could quantify ATMS and that strengthening exercises using the device increased ATMS and activated the abdominals, diaphragmatic, and pelvic floor muscles [22]. Muscle contraction during the ATMS measurement and the strengthening exercise was comparable with that involved with abdominal bracing when the abdominal and paraspinal muscles were activated [28]. The abdominal core can be described as a muscular box with the abdominals at the front and sides, the paraspinals at the back, the diaphragm at the roof, and the pelvic floor at the bottom of the box [29]. The contraction of the diaphragm increased intra-abdominal pressure and stabilized the spine [29]. ATMS is created through the coordinated contraction of the trunk muscles comprising the muscular box. This muscle contraction creates a semirigid cylinder surrounding the spinal column with increased intra-abdominal pressure, reducing some of the imposed stress on the vertebral column in the lower thoracic and lumbar spine. However, study results remain inconclusive due to a lack of consensus concerning how core strength is measured [30]. If core strength and stability could be easily and reliably measured, the physical condition of the patient could be more accurately determined, individuals requiring core muscle strengthening could be identified, and therapeutic intervention could be more appropriate. This device may be a viable option for measuring core muscle strength and can potentially evaluate core instability associated with a future risk of OVF. The ROC analysis in this study indicated that AMTS ≤ 4.0 kPa was related to a risk of OVF occurrence in middle-aged and older adult women. Thus, ATMS measurements can be used to assess the risk of OVF. A previous study has reported that strengthening exercise using the device improved ATMS and mobility function, assessed using the stand-up test in an older adult population [31]. The coordinated contraction of abdominal trunk muscles with increased intra-abdominal pressure helps to perform the action of standing up. A recent study has reported that a poor stand-up test score was an independent risk factor for non-traumatic OVF occurrence [16]. This result indirectly indicated that trunk stability and function, evaluated as ATMS, could be an important indicator of OVF risk.

Recent studies investigating spinal sagittal imbalance have reported that a large sagittal vertical axis was a risk factor for future OVF or OVF collapse [16,17,32]. Patients in the fracture group had a smaller sacral slope and larger pelvic tilt than those in the non-fracture group; however, the sagittal vertical axis did not differ between the groups. A small sacral slope and a large pelvic tilt indicate pelvic retroversion associated with sagittal imbalance [25]. However, in the multivariate analysis, these parameters were not identified as risk factors for new OVF. In our study cohort, trunk muscle strength was observed to be more important than spinal sagittal imbalance as an indicator of OVF risk. Previous studies have reported that back extensor strength is an important factor affecting spinal deformity or alignment and quality of life in middle-aged and older adult women [33,34,35]. Sinaki et al. [36] reported that postmenopausal women who engaged in back extensor strengthening had a lower occurrence of future OVF. Thus, trunk extension exercises with isometric muscle contraction have been considered appropriate for middle-aged and older adult women with osteoporosis [37,38]. In muscle strength measurements and strengthening exercises using the device in this study, abdominal trunk muscles, including diaphragmatic, abdominal, and pelvic floor muscles, were activated without the need for trunk movement. Such isometric muscle contraction is safe and appropriate for fragile spines in older patients with osteoporosis or low back pain. Therefore, using the device as a strengthening exercise may also be useful in improving the physical function of patients with age-related musculoskeletal disorders, including osteoporosis, and preventing the occurrence of OVF, particularly for patients with weak ATMS.

This study had some limitations. First, this study included a small number of participants with new OVFs. Second, only patients undergoing surgery for degenerative disease of the lower extremities were analyzed, which might have influenced the data on lower KEMSs, higher L-BMDs, and altered spinopelvic alignment findings. Finally, back extensor strength, which has been reported as a key muscle strength for spinal alignment or future OVF [34,35,36], was not measured. Although this study measured functional muscle parameters including hand grip strength, KEMS, and ATMS, body composition measurements, such as muscle and fat mass, were not performed. Future studies are required to examine whether abdominal trunk muscle weakness increases future OVF occurrence and whether abdominal trunk muscle strengthening reduces future OVF occurrence in a larger number of healthy volunteers without musculoskeletal diseases; this is to compare the effect of weak abdominal trunk muscles versus back extensor muscles and the efficacy of strengthening these muscles.

## 5. Conclusions

Abdominal trunk muscle weakness, low L-BMD, and the presence of old OVFs were significant risk factors associated with OVF occurrence in the lower thoracic or lumbar spine. ATMS measurement can be used to assess the risk of future OVF occurrence.

## Figures and Tables

**Table 1:** Baseline characteristics of the patients with and without the second examination.

	Patients with the 2nd Examination	Patients without the 2nd Examination	*p*-Value
**No. of participants**	143	54	
**Age (years), mean ± SD [range]**	65.8 ± 8.3 (50–84)	69.1 ± 7.8 (53–84)	0.013
**BMI (kg/m^2^), mean ± SD [range]**	23.8 ± 3.9 (14.7–36.7)	24.4 ± 4.8 (14.1–34.4)	0.411
**L-BMD (g/cm^2^), mean ± SD [range]**	1.07 ± 0.22 (0.63–1.80)	1.01 ± 0.18 (0.65–1.37)	0.057
**Presence of old OVF, no. (%)**	17 (11.9)	7 (13.0)	0.837
**Disease pathology (n)**	Hip joint disease (100) Knee joint disease (20) Foot & ankle disease (23)	Hip joint disease (35) Knee joint disease (8) Foot & ankle disease (11)	

BMI, body mass index; L-BMD, bone mineral density of the lumbar spine; OVF, osteoporotic vertebral fracture; SD, standard deviation.

**Table 2:** Differences in the baseline characteristics between the fracture and non-fracture groups.

	Fracture Group	Non-Fracture Group	*p*-Value
**No. of subjects**	10	131	
**Age (years), mean ± SD**	67.5 ± 9.3	65.6 ± 8.3	0.491
**BMI (kg/m^2^), mean ± SD**	25.4 ± 5.7	23.7 ± 3.7	0.177
**Hand grip strength (kg), mean ± SD**	18.3 ± 6.1	21.1 ± 5.0	0.089
**KEMS (N/kg), mean ± SD**	3.5 ± 1.6	3.8 ± 1.2	0.552
**ATMS (kPa), mean ± SD**	2.7 ± 1.8	5.3 ± 2.8	0.006
**GLFS-25 score (point), mean ± SD**	48.0 ± 27.2	40.0 ± 19.3	0.221
**NRS (0–4) of back pain (point), median [IQR]**	1 [1–1]	1 [0–2]	0.431
**L-BMD (g/cm^2^), mean ± SD**	0.93 ± 0.16	1.08 ± 0.22	0.027
**Presence of old OVF, No. (%)**	6 (60)	11 (8.4)	<0.001
**Sagittal vertical axis (mm), mean ± SD**	51.1 ± 39.9	38.8 ± 42.6	0.378
**Lumbar lordosis (degree), mean ± SD**	43.7 ± 19.6	46.1 ± 16.7	0.671
**Pelvic incidence (degree), mean ± SD**	52.8 ± 9.0	55.4 ± 10.6	0.451
**Sacral slope (degree), mean ± SD**	27.8 ± 13.9	38.4 ± 12.3	0.010
**Pelvic tilt (degree), mean ± SD**	24.7 ± 10.7	17.0 ± 11.1	0.036
**Pelvic incidence minus Lumbar lordosis (degree), mean ± SD**	9.1 ± 15.1	9.4 ± 15.8	0.962

ATMS, abdominal trunk muscle strength; BMI, body mass index; GLFS-25, 25-Question Geriatric Locomotive Function Scale; IQR, interquartile range; KEMS, knee extensor muscle strength; L-BMD, bone mineral density of the lumbar spine; NRS, numerical rating scale; OVF, osteoporotic vertebral fracture; SD, standard deviation.

**Table 3:** Multivariate analysis of factors associated with the occurrence of new OVFs in the lower thoracic or lumbar spine.

	Reference	aOR	*p*-Value	95% CI
**ATMS (kPa)**	+1 kPa	0.557	0.037	0.322–0.964
**L-BMD (g/cm^2^)**	+1 SD	0.226	0.011	0.072–0.707
**Presence of old OVF**	No old OVF	6.956	0.023	1.304–37.105
**Sacral slope (degree)**	+1 kPa	0.924	0.087	0.843–1.012
**Pelvic tilt (degree)**	+1 kPa	0.973	0.588	0.882–1.073

aOR; adjusted odds ratio; ATMS, abdominal trunk muscle strength; CI, confidence interval; L-BMD, bone mineral density of the lumbar spine; OVF, osteoporotic vertebral fracture.

## Data Availability

The datasets used and analyzed during the current study are available from the corresponding author upon reasonable request.

## References

[1] Burge R., Dawson-Hughes B., Solomon D.H., Wong J.B., King A., Tosteson A. (2007). Incidence and economic burden of osteoporosis-related fractures in the United States, 2005–2025. J. Bone Miner. Res..

[2] Christiansen B.A., Bouxsein M.L. (2010). Biomechanics of vertebral fractures and the vertebral fracture cascade. Curr. Osteoporos. Rep..

[3] Lindsay R., Silverman S.L., Cooper C., Hanley D.A., Barton I., Broy S.B., Licata A., Benhamou L., Geusens P., Flowers K. (2001). Risk of new vertebral fracture in the year following a fracture. JAMA.

[4] Horii C., Asai Y., Iidaka T., Muraki S., Oka H., Tsutsui S., Hashizume H., Yamada H., Yoshida M., Kawaguchi H. (2019). Differences in prevalence and associated factors between mild and severe vertebral fractures in Japanese men and women: The third survey of the ROAD study. J. Bone Miner. Metab..

[5] Kado D.M., Duong T., Stone K.L., Ensrud K.E., Nevitt M.C., Greendale G.A., Cummings S.R. (2003). Incident vertebral fractures and mortality in older women: A prospective study. Osteoporos. Int..

[6] Jalava T., Sarna S., Pylkkänen L., Mawer B., Kanis J.A., Selby P., Davies M., Adams J., Francis R.M., Robinson J. (2003). Association between vertebral fracture and increased mortality in osteoporotic patients. J. Bone Miner. Res..

[7] Schousboe J.T. (2016). Epidemiology of Vertebral Fractures. J. Clin. Densitom..

[8] Cooper C., O’Neill T., Silman A. (1993). The epidemiology of vertebral fractures. European Vertebral Osteoporosis Study Group. Bone.

[9] Balasubramanian A., Zhang J., Chen L., Wenkert D., Daigle S.G., Grauer A., Curtis J.R. (2019). Risk of subsequent fracture after prior fracture among older women. Osteoporos. Int..

[10] Banefelt J., Åkesson K.E., Spångéus A., Ljunggren O., Karlsson L., Ström O., Ortsäter G., Libanati C., Toth E. (2019). Risk of imminent fracture following a previous fracture in a Swedish database study. Osteoporos. Int..

[11] Mitchell W.K., Williams J., Atherton P., Larvin M., Lund J., Narici M. (2012). Sarcopenia, dynapenia, and the impact of advancing age on human skeletal muscle size and strength; a quantitative review. Front. Physiol..

[12] Delmonico M.J., Harris T.B., Visser M., Park S.W., Conroy M.B., Velasquez-Mieyer P., Boudreau R., Manini T.M., Nevitt M., Newman A.B. (2009). Longitudinal study of muscle strength, quality, and adipose tissue infiltration. Am. J. Clin. Nutr..

[13] Singh D.K., Bailey M., Lee R.Y. (2011). Ageing modifies the fibre angle and biomechanical function of the lumbar extensor muscles. Clin Biomech (Bristol, Avon).

[14] Anderson D.E., D’Agostino J.M., Bruno A.G., Demissie S., Kiel D.P., Bouxsein M.L. (2013). Variations of CT-based trunk muscle attenuation by age, sex, and specific muscle. J. Gerontol. A Biol. Sci. Med. Sci..

[15] Dai J., Yu X., Huang S., Fan L., Zhu G., Sun H., Tang X. (2015). Relationship between sagittal spinal alignment and the incidence of vertebral fracture in menopausal women with osteoporosis: A multicenter longitudinal follow-up study. Eur. Spine J..

[16] Asahi R., Nakamura Y., Kanai M., Watanabe K., Yuguchi S., Kamo T., Azami M., Ogihara H., Asano S. (2021). Stand-up test predicts occurrence of non-traumatic vertebral fracture in outpatient women with osteoporosis. J. Bone Miner. Metab..

[17] Lin T., Lu J., Zhang Y., Wang Z., Chen G., Gu Y., Zhou L., Liu W. (2021). Does spinal sagittal imbalance lead to future vertebral compression fractures in osteoporosis patients. Spine J..

[18] Dreischarf M., Shirazi-Adl A., Arjmand N., Rohlmann A., Schmidt H. (2016). Estimation of loads on human lumbar spine: A review of in vivo and computational model studies. J. Biomech..

[19] Kato S., Demura S., Kurokawa Y., Shinmura K., Yokogawa N., Yonezawa N., Shimizu T., Oku N., Kitagawa R., Matsubara H. (2019). Correlation between osteoporotic vertebral fracture and abdominal trunk muscle strength in middle-aged and older women. Arch. Osteoporos..

[20] Suzuki T. (2015). Reliability of measurements of knee extensor muscle strength using a pull-type hand-held dynamometer. J. Phys. Ther. Sci..

[21] Kato S., Murakami H., Inaki A., Mochizuki T., Demura S., Nakase J., Yoshioka K., Yokogawa N., Igarashi T., Takahashi N. (2017). Innovative exercise device for the abdominal trunk muscles: An early validation study. PLoS ONE.

[22] Kato S., Inaki A., Murakami H., Kurokawa Y., Mochizuki T., Demura S., Yoshioka K., Yokogawa N., Yonezawa N., Shimizu T. (2020). Reliability of the muscle strength measurement and effects of the strengthening by an innovative exercise device for the abdominal trunk muscles. J. Back Musculoskelet. Rehabil..

[23] Nakamura K., Ogata T. (2016). Locomotive Syndrome: Definition and Management. Clin. Rev. Bone Miner. Metab..

[24] Genant H.K., Wu C.Y., van Kuijk C., Nevitt M.C. (1993). Vertebral fracture assessment using a semiquantitative technique. J. Bone Miner. Res..

[25] Schwab F., Ungar B., Blondel B., Buchowski J., Coe J., Deinlein D., DeWald C., Mehdian H., Shaffrey C., Tribus C. (2012). Scoliosis Research Society-Schwab adult spinal deformity classification: A validation study. Spine (Phila Pa 1976).

[26] Wu J., Wei F., Ma L., Li J., Zhang N., Tian W., Sun Y. (2021). Accuracy and Reliability of Standing Lateral Lumbar Radiographs for Measurements of Spinopelvic Parameters. Spine (Phila Pa 1976).

[27] Inose H., Kato T., Ichimura S., Nakamura H., Hoshino M., Togawa D., Hirano T., Tokuhashi Y., Ohba T., Haro H. (2021). Risk factors for subsequent vertebral fracture after acute osteoporotic vertebral fractures. Eur. Spine J..

[28] Kurokawa Y., Kato S., Demura S., Shinmura K., Yokogawa N., Yonezawa N., Shimizu T., Kitagawa R., Miaki H., Tsuchiya H. (2022). Validation and comparison of trunk muscle activities in male participants during exercise using an innovative device and abdominal bracing maneuvers. J. Back Musculoskelet. Rehabil..

[29] Akuthota V., Ferreiro A., Moore T., Fredericson M. (2008). Core stability exercise principles. Curr. Sports Med. Rep..

[30] Akuthota V., Nadler S.F. (2004). Core strengthening. Arch. Phys. Med. Rehabil..

[31] Kato S., Demura S., Kurokawa Y., Takahashi N., Shinmura K., Yokogawa N., Yonezawa N., Shimizu T., Kitagawa R., Tsuchiya H. (2020). Efficacy and Safety of Abdominal Trunk Muscle Strengthening Using an Innovative Device in Elderly Patients with Chronic Low Back Pain: A Pilot Study. Ann. Rehabil. Med..

[32] Ohnishi T., Iwata A., Kanayama M., Oha F., Hashimoto T., Iwasaki N. (2018). Impact of spino-pelvic and global spinal alignment on the risk of osteoporotic vertebral collapse. Spine Surg. Relat. Res..

[33] Hongo M., Itoi E., Sinaki M., Miyakoshi N., Shimada Y., Maekawa S., Okada K., Mizutani Y. (2007). Effect of low-intensity back exercise on quality of life and back extensor strength in patients with osteoporosis: A randomized controlled trial. Osteoporos. Int..

[34] Hongo M., Miyakoshi N., Shimada Y., Sinaki M. (2012). Association of spinal curve deformity and back extensor strength in elderly women with osteoporosis in Japan and the United States. Osteoporos. Int..

[35] Miyakoshi N., Kudo D., Hongo M., Kasukawa Y., Ishikawa Y., Shimada Y. (2017). Comparison of spinal alignment, muscular strength, and quality of life between women with postmenopausal osteoporosis and healthy volunteers. Osteoporos. Int..

[36] Sinaki M., Itoi E., Wahner H.W., Wollan P., Gelzcer R., Mullan B.P., Collins D.A., Hodgson S.F. (2002). Stronger back muscles reduce the incidence of vertebral fractures: A prospective 10 year follow-up of postmenopausal women. Bone.

[37] Sinaki M., Mikkelsen B.A. (1984). Postmenopausal spinal osteoporosis: Flexion versus extension exercises. Arch. Phys. Med. Rehabil..

[38] Sinaki M. (2012). Exercise for patients with osteoporosis: Management of vertebral compression fractures and trunk strengthening for fall prevention. PM R.

